# The Heisenberg Indeterminacy Principle in the Context of Covariant Quantum Gravity

**DOI:** 10.3390/e22111209

**Published:** 2020-10-26

**Authors:** Massimo Tessarotto, Claudio Cremaschini

**Affiliations:** 1 Department of Mathematics and Geosciences, University of Trieste, Via Valerio 12, 34127 Trieste, Italy; maxtextss@gmail.com; 2 Research Center for Theoretical Physics and Astrophysics, Institute of Physics, Silesian University in Opava, Bezručovo nám.13, CZ-74601 Opava, Czech Republic

**Keywords:** covariant quantum gravity, Heisenberg indeterminacy principle, Heisenberg inequalities, quantum probability density function, deterministic limit

## Abstract

The subject of this paper deals with the mathematical formulation of the Heisenberg Indeterminacy Principle in the framework of Quantum Gravity. The starting point is the establishment of the so-called time-conjugate momentum inequalities holding for non-relativistic and relativistic Quantum Mechanics. The validity of analogous Heisenberg inequalities in quantum gravity, which must be based on strictly physically observable quantities (i.e., necessarily either 4-scalar or 4-vector in nature), is shown to require the adoption of a manifestly covariant and unitary quantum theory of the gravitational field. Based on the prescription of a suitable notion of Hilbert space scalar product, the relevant Heisenberg inequalities are established. Besides the coordinate-conjugate momentum inequalities, these include a novel proper-time-conjugate extended momentum inequality. Physical implications and the connection with the deterministic limit recovering General Relativity are investigated.

## 1. Introduction

In this paper, a basic challenge in theoretical and mathematical physics is addressed which concerns the axiomatic approach to Quantum Gravity and has fundamental implications in relativistic astrophysics, quantum gravity theory, and cosmology. The issue is about the identification of some general theoretical prerequisites for setting up—in a proper sense—a quantum theory of the gravitational field, i.e., Quantum Gravity (QG). In particular, these concern two closely-related physical requirements:

1. The first one deals with the notion of quantum uncertainty in the context of Quantum Gravity. In detail, this refers to the identification of the (possible) conditions of validity of the so-called Heisenberg indeterminacy principle (HIP), i.e., the prescription of suitable Heisenberg inequalities (also referred to in the literature as “uncertainty relations”) which should hold in this context. The conjecture which is advanced here is that these should be in some sense analogous to those which apply in the context of both non-relativistic and relativistic Quantum Mechanics (QM).
2. The second one concerns the logical connection with classical physics. In particular, it is about the relationship of the Heisenberg indeterminacy principle with the Deterministic Principle which applies in the context of General Relativity (GR).

The first topic deals, more precisely, with the identification of the appropriate sets of Heisenberg inequalities holding in the context of QG and the conditions of validity (if any) to be set on the quantum-wave function and related quantum observables for the same uncertainty principle to apply. In fact, in analogy with ordinary QM [1], one should expect that different types of inequalities should actually hold for different sets of canonically conjugate variables. In the case of QG, the subject has been initially investigated in [2], where tensor Heisenberg inequalities associated with conjugate-canonical variables of the unitary representation of the manifestly covariant quantum gravity theory (CQG theory) based on the works in [3,4,5,6] were achieved. However, one expects that, in principle, an additional Heisenberg inequality should also apply in the same framework, similar to the one holding in QM for the couple of variables represented by absolute time and its extended canonical conjugate momentum, previously identified with energy. The second topic instead involves the determination of the asymptotic conditions—to be set both on the solution of the quantum-gravity probability density function (PDF) and the corresponding quantum observables—in such a way to warrant that the deterministic limit applying in classical GR is actually recovered.

In connection with this, basic questions about the fundamental basis for quantum gravity theories arise. The first one concerns, in fact, the mandatory physical requirements to be set on the theory of quantum gravity itself as a consequence of HIP, with particular reference to the tensor properties which the relevant dynamical variables should exhibit. Another question concerns the conditions of application of the indeterminacy principle with respect to the solution of the quantum-gravity wave function and the global validity of the Heisenberg inequalities for arbitrary quantum PDFs. In fact, it is not a priori obvious that HIP should actually hold for an arbitrary quantum wave function to be associated with the space-time quantum state, i.e., which is a physically realizable solution of the relevant quantum-wave equation. In particular, the issue is whether HIP applies in the case of Gaussian solutions for the quantum PDF which holds in the context of unitary formulation of QG [7,8].

The construction of explicit solutions of the CQG quantum-wave equation is a basic prerequisite for the task posed in this paper. This is based here on the original formulation of CQG theory given in [5,6] (see also in [2]), i.e., in the framework of a unitary representation for a continuous and manifestly covariant quantum theory of the gravitational field. This follows from the Hamilton–Jacobi quantization (*g*-quantization) of a classical continuous Hamiltonian system associated with the Einstein field equations (EFE) of GR [9,10]. A crucial property of such a classical Hamiltonian structure underlying EFE is that of being constraint-free and manifestly covariant when expressed in suitable superabundant canonical variables. The above quantization is unitary in character because the effect of possible quantum sources and sinks (leading respectively to creation and destruction of massive gravitons) is expected to be highly localized, as these events should presumably occur very close to black hole event horizons [11,12]. Therefore, for this reason they can be safely neglected at least in first formulation of the theory.

The purpose of the investigation is to extend the Heisenberg Indeterminacy Principle established in [2]. More precisely, we intend to show that besides the customary case of canonically conjugate variables represented by Lagrangian coordinates and their conjugate quantum canonical momenta, a further Heisenberg inequality exists, which is analogous to the well-known inequality valid in the case of the coordinate time (respectively, proper time), and the corresponding conjugate extended quantum momentum which hold in non relativistic (respectively, relativistic) quantum mechanics. Finally, the conditions of validity of the resulting Heisenberg inequalities, together with their deterministic limits are discussed, and the main conclusions of the present investigation are drawn.

To deal with these issues in a rigorous fashion, however, requires changing the native definition of the Hilbert space scalar product. As we intend to show the procedure is unusual but mathematically sound. However, to better understand how technically this can be achieved in practice and to grasp its actual physical motivations, in the following the same problem is first posed in the context of both non-relativistic and relativistic quantum mechanics (NRQM and RQM), where a rigorous solution of the analogous problem is actually possible.

## 2. The Heisenberg Uncertainty Principle

It is well known that the Heisenberg indeterminacy principle (HIP) represents a fundamental ingredient for ordinary QM and quantum field theory. In fact, in some sense, its validity can be viewed as a paradigm of quantum theory in itself. The indeterminacy arises because of the concept of quantum measurement and the non-deterministic nature of quantum observables. This means that their quantum expectation value must be intrinsically non deterministic, i.e., characterized by a non-vanishing standard deviation. In accordance with HIP, in the case of suitable sets of conjugate quantum variables/operators, one therefore expects that the product of the corresponding quantum standard deviations should be always non-vanishing. Specifically, this occurs because in quantum field theory, just as QM, it is always possible to introduce quantum canonical variables, i.e., generalized Lagrangian coordinates and conjugate-momentum operators generally represented by suitable tensor fields/operators, in terms of which all the quantum observables of the system are prescribed. This feature has the mandatory consequence that indeterminacy affects not just the quantum canonical variables themselves but all quantum observables. Therefore, it is understandable why in the theoretical and mathematical physics communities alike it is commonly agreed that the validity of HIP should be at the basis of any field theory to be denoted as “quantum”.

### 2.1. The Case of NRQM

Although the notion of HIP is undoubtedly deeply rooted to the foundations of QM, nevertheless certain aspects require clarification. For definiteness, it is instructive to recall, first, the realization of HIP in the context of non-relativistic quantum mechanics (NRQM) for which the appropriate notations are recalled in Appendix A. In such a case, the Heisenberg principle follows as an immediate consequence of the generalized Heisenberg uncertainty relations:

(1) $$\langle \psi \left|{\left(A-\bar{A}\right)}^{2}\psi \right.\rangle \langle \psi \left|{\left(B-\bar{B}\right)}^{2}\psi \right.\rangle \geq {\left|\frac{1}{2i}\langle \psi \left|{\left[A,B\right]}^{\left(q\right)}\psi \right.\rangle \right|}^{2},$$

where *A* and *B* denote two arbitrary Hermitian operators and ${\left[A,B\right]}^{\left(q\right)}=AB-BA$ is their quantum commutator. This form of the Heisenberg principle is well known and was obtained in 1929 by Robertson [13] following the original work by Heisenberg [14] (for this reason Equation (1) is also known as *Robertson uncertainty relation*). Let us consider for this purpose the Schroedinger equation:

(2) $$i\hbar \frac{\partial \psi (r,t)}{\partial t}=H\psi \left(r,t\right),$$

with $H=\frac{\hbar^{2}}{2m}\nabla_{r}^{2}+V\left(r,t\right)$ being the Hamiltonian operator, V(r,t) a smooth classical potential, and the quantum wave-function ψ(r,t) belonging to a Hilbert space (which requires the introduction of a suitable scalar product). In the standard formulation of NRQM, for arbitrary functions $\psi_{a}\left(r,t\right)$ and $\psi_{b}\left(r,t\right)$ of the same Hilbert space, the customary realization of the scalar product (due originally to Heisenberg himself) is provided by the so-called *local scalar product*, namely,

(3) $$\langle \psi_{a}|\psi_{b}\rangle \equiv \int_{\Omega }d^{3}r\psi_{a}^{*}\left(r,t\right)\psi_{b}\left(r,t\right),$$

with $d\omega =d^{3}r$ being the volume element of the Euclidean configuration space Ω (i.e., properly a domain isomorphic or coinciding with the Euclidean space on $R^{3}$). In the case in which *H* is Hermitian (i.e., the case considered here), this implies by construction that denoting $\psi \left(r,t\right)\equiv \psi_{b}\left(r,t\right)$ an arbitrary function of the Hilbert space (representing an arbitrary particular solution of the Schroedinger Equation (2)), it must be $\langle \psi |\psi \rangle \equiv \int_{\Omega }d^{3}r\rho \left(r,t\right)=1$, where ${\left|\psi (r,t)\right|}^{2}=\rho \left(r,t\right)>0$ is therefore a probability density on the 3-dimensional Euclidean configuration space Ω.

Based on the prescription (3) and on the Robertson inequality (1) it is then immediate to draw some basic implications and recover also the famous *position-conjugate momentum Heisenberg inequalities*. First one notices, in fact, that if one denotes by $\bar{A}$ and $\bar{B}$ the quantum expectation values $\bar{A}=\langle \psi \left|A\psi \right.\rangle$ and $\bar{B}=\langle \psi \left|B\psi \right.\rangle$, then validity of Robertson inequality (1) demands that the same function ψ cannot be an eigenfunction of either *A* or *B*. In fact, if, for example, $A\psi =\bar{A}\psi$, then it follows $\langle \psi \left|{\left(A-\bar{A}\right)}^{2}\psi \right.\rangle =\langle \psi \left|\left(A^{2}-2\bar{A}A+{\bar{A}}^{2}\right)\psi \right.\rangle =0$. Second, let us identify the operators *A* and *B* so that $A=r^{i}$ (the Cartesian components of the position 3-vector $r\equiv \left\{r^{i}\right\}$ which spans the Euclidean space Ω on $R^{3}$, or more generally arbitrary Lagrangian coordinates) and, respectively, $B=\pi_{r^{i}}^{\left(q\right)}$, with $\pi_{r^{i}}^{\left(q\right)}$ for i=1,2,3 being their conjugate quantum canonical momentum operators

(4) $$\pi_{i}^{\left(q\right)}\equiv -i\hbar \frac{\partial }{\partial r^{i}}.$$

Then, it is immediate to show that the Robertson inequality (1) implies the *position-momentum* (or *conjugate-canonical variables* in case $r^{i}$ for i=1,2,3 denote arbitrary Lagrangian coordinates) *Heisenberg inequalities*

(5) $$\sigma_{r^{\left(i\right)}}\sigma_{\pi_{\left(i\right)}^{\left(q\right)}}\geq \frac{\hbar }{2}.$$

Notice that this set of inequalities (discovered by Heisenberg himself in 1927) can actually be regarded as a *single* 3-*vector inequality*, as it holds separately for each vector component ($r^{i},\pi_{i}^{\left(q\right)}$), i.e., for i=1,2,3. Notice that here $\sigma_{r^{i}}$ and $\sigma_{\pi_{i}^{\left(q\right)}}$ are the standard deviations defined as usual in terms of the variances, i.e., $\sigma_{r^{i}}^{2}=\langle \psi \left|{\left(r^{i}-{\bar{r}}^{i}\right)}^{2}\psi \right.\rangle$ and $\sigma_{\pi_{i}^{\left(q\right)}}^{2}=\langle \psi \left|{\left(\pi_{i}^{\left(q\right)}-\bar{\pi_{i}^{\left(q\right)}}\right)}^{2}\psi \right.\rangle$, with $\left({\bar{r}}^{i},\bar{\pi_{i}^{\left(q\right)}}\right)$ being the quantum expectation values ${\bar{r}}^{i}=\langle \psi |r^{i}\psi \rangle$ and $\bar{\pi_{i}^{\left(q\right)}}=\langle \psi \left|\pi_{i}^{\left(q\right)}\psi \right.\rangle$. The proof of (5) follows as a consequence of the canonical commutation rule

(6) $${\left[r^{i},\pi_{j}^{\left(q\right)}\right]}^{\left(q\right)}=-i\hbar \delta_{j}^{i}.$$

However, there is a fourth (scalar) characteristic Heisenberg inequality in NRQM, which is associated with canonically conjugate quantum variables (A=t,$B=\pi_{t}^{\left(q\right)}\equiv -i\hbar \frac{\partial }{\partial t})$, with *t* being the absolute time and $\pi_{t}^{\left(q\right)}$ its canonically conjugate quantum operator. Notice that this case differs from the so called time-energy uncertainty relation usually considered in the literature and at the same time one of the most controversial formulas of quantum theory. The usual objection to the time-energy inequality due to Pauli [15] (see also in [16,17,18]) is that it should not be possible to define the quantum commutator ${\left[t,H\right]}^{\left(q\right)}$, where *t* is interpreted as time. However, the claimed impossibility of defining the quantum commutator does not apply to the variables $\left(t,\pi_{t}^{\left(q\right)}\right)$, with *t* being intended as an operator. The reason is very clear. In fact, the commutation rule

(7) $${\left[t,\pi_{t}^{\left(q\right)}\right]}^{\left(q\right)}\equiv t\pi_{t}^{\left(q\right)}-\pi_{t}^{\left(q\right)}t=i\hbar$$

must hold by assumption thanks to the Schroedinger equation. This follows in an elementary way by applying the Hamilton–Jacobi (HJ) canonical quantization rule. Therefore, in the present case the Pauli objection does not apply. Despite this feature, the Richardson inequality cannot be invoked because both *t* and $\pi_{t}^{\left(q\right)}$ are obviously non-Hermitian with respect to the local scalar product (3), so the proof cannot be reached in this way.

The proof of a time-energy uncertainty relation of the form

(8) $$\sigma_{t}\sigma_{H}\geq \frac{\hbar }{2},$$

was nonetheless achieved by Mandelstam and Tamm in 1945 [19], with $\sigma_{H}$ being the standard deviation such that $\sigma_{H}^{2}=\langle \psi \left|{\left(H-\bar{H}\right)}^{2}\psi \right.\rangle$ (with $\bar{H}$ being the expectation value $\bar{H}=\langle \psi \left|H\psi \right.\rangle$) and $\sigma_{t}$ denoting a suitably-defined time uncertainty. Mandelstam and Tamm in fact found a way to represent δt, which actually they interpreted as *the (minimum) quantum measurement time for the quantum system*, i.e., the minimum time to experience a meaningful change among all the observables of the quantum system. Such a result does not require any modification of the postulates of Standard Quantum Mechanics (SQM).

In view of the previous considerations, however, another route is available for the conjugate quantum variables (A=t,$B=\pi_{t}^{\left(q\right)}\equiv -i\hbar \frac{\partial }{\partial t})$ for the purpose of reaching for them a rigorous proof of a Heisenberg-type uncertainty relation. This is formally achieved by seeking a new definition of the scalar product (replacing Equation (3)) such that both *t* and $\pi_{t}^{\left(q\right)}$ become Hermitian operators with respect to it. It is immediate to realize that such a definition is actually provided by the *time-averaged scalar product*

(9) $$\langle \langle \psi_{a}|\psi_{b}\rangle \rangle \equiv \frac{\int_{t_{o}}^{t_{o}+\Delta t}dt\int_{\Omega }d\omega \psi_{a}^{*}\left(r,t\right)\psi_{b}\left(r,t\right)}{\Delta t},$$

with Δt denoting a still arbitrary amplitude of the time interval referred to as *quantum measurement time for the quantum system,* to be intended as a suitable time interval in which the quantum measurement is performed. That this is indeed a scalar product follows by noting that, by letting $\psi_{a}^{*}\left(r,t\right)=\psi^{*}\left(r,t\right)$, $\psi_{b}\left(r,t\right)=\psi \left(r,t\right)$ with ψ(r,t) denoting an arbitrary element of the function space (such as, for example, a particular solution of the Schroedinger Equation (2)), again by construction the equation $\langle \langle \psi |\psi \rangle \rangle =1$ holds identically. Thus, it is obvious that, once the Hilbert space associated with the same equation is defined with respect to the local scalar product (3), it follows that it can also be equivalently prescribed in terms of the non-local one (9). However, in this way as a notable consequence the said Hermitian property is warranted by construction for *t* because manifestly $\langle \langle \psi |t\psi \rangle \rangle =\langle \langle t\psi |\psi \rangle \rangle$.Similarly, $\langle \langle \psi |\pi_{t}^{\left(q\right)}\psi \rangle \rangle =\langle \langle \pi_{t}^{(q)*}\psi |\psi \rangle \rangle$ because integration by parts yields

(10) $$\langle \langle \psi \left|\left(-i\hbar \right)\frac{\partial }{\partial t}\psi \right.\rangle \rangle =\frac{-i\hbar }{\Delta t}{\left[\int_{\Omega }d\omega {\left|\psi^{*}\left(r,t\right)\right|}^{2}\right]}_{t_{o}}^{t_{o}+\Delta t}+\langle \langle \left.\left(-i\hbar \right)\frac{\partial }{\partial t}\psi \right|\psi \rangle \rangle ,$$

while the first term on the rhs vanishes identically (as $\int_{\Omega }d\omega {\left|\psi^{*}\left(r,t\right)\right|}^{2}=1$). Thus, the modified scalar product (9) permits to reach the desired conclusion by simple application of the Robertson inequality (1). Denoting by $\bar{t}=\langle \langle \psi \left|t\psi \right.\rangle \rangle$ and $\bar{\pi_{t}^{\left(q\right)}}=\langle \langle \psi \left|\pi_{t}^{\left(q\right)}\psi \right.\rangle \rangle$ the corresponding quantum expectation values and noting that $\langle \langle \psi \left|\left[t,\pi_{t}^{\left(q\right)}\right]\psi \right.\rangle \rangle =i\hbar$, the so-called time-extended canonical momentum Heisenberg inequality follows,

(11) $$\sigma_{t}\sigma_{\pi_{t}^{\left(q\right)}}\geq \frac{\hbar }{2},$$

with $\sigma_{t}$ and $\sigma_{\pi_{t}^{\left(q\right)}}$ being now the standard deviations evaluated in terms of scalar product (9), i.e., such that $\sigma_{t}^{2}=\langle \langle \psi \left|{\left(t-\bar{t}\right)}^{2}\psi \right.\rangle \rangle$ and $\sigma_{\pi_{t}^{\left(q\right)}}^{2}=\langle \langle \psi \left|{\left(\pi_{t}^{\left(q\right)}-\bar{\pi_{t}^{\left(q\right)}}\right)}^{2}\psi \right.\rangle \rangle$.

It is obvious that the introduction of the non-local scalar product (9) represents a departure from the axiom of locality of SQM. This conclusion may look surprising. However, the following remarks are in order.

- First, we have shown that the prescription (9) is physically motivated, i.e., by the requirement that both time *t*, treated as a quantum operator, and its conjugate quantum momentum operator $\pi_{t}^{\left(q\right)}$ are Hermitian (which means that $\pi_{t}^{\left(q\right)}$ becomes an observable).
- Second, the notion of *quantum measurement time for a quantum system* which is attached to the prescription of the time-averaged scalar product given above is actually not new (at least in qualitative sense), being due already to Mandelstam and Tamm [19].
- Third, the interesting implication of the time-extended momentum Heisenberg uncertainty relation (11) is that the minimum value of Δt is not arbitrary, being actually prescribed by the same inequality. In fact, straightforward algebra delivers that $\sigma_{t}\equiv \frac{\Delta t}{2\sqrt{3}}$, which means that $\Delta t\sigma_{\pi_{t}^{\left(q\right)}}\geq \sqrt{3\hbar }$.
- Fourth, the introduction of the non-local scalar product (9) does not affect the definition of the Hilbert space associated with the quantum system, which remains instead, as usual, uniquely associated with the customary local definition (3), thus warranting its completeness property.

### 2.2. The Extension to RQM

A generalization of the two sets of Heisenberg inequalities (5) and (11) can be achieved in the context of relativistic quantum mechanics. For definiteness, let us consider here the relativistic quantum description for extended charged spinless particles subject to the action of the electromagnetic (EM) radiation-reaction (RR) interaction. In the following, we adopt a working framework known as the so-called single-particle approach, namely, assuming existence of a single relativistic particle for which the corresponding 1-body Schroedinger equation in unitary form is given. Although the single-particle formulation might still be characterized by the occurrence of problems like micro-causality violations (arising due to loss of unitarity), in contrast to the case of a multi-particle approach, the mathematical formulation of the corresponding quantum dynamics is instructive for the understanding of the subsequent formalism to be established in quantum gravity theory. More precisely, as shown in [20] in this case a trajectory-based relativistic quantum wave equation can be achieved in which quantization pertains to particle dynamics while both the external and self EM fields can be treated classically. Accordingly, the so-called *radiation reaction (RR) quantum wave equation* takes the form of a relativistic Schroedinger equation of the type

(12) $$i\hbar \frac{\partial }{\partial s}\psi \left(r,s\right)=\hat{H}\left(r,\hat{\pi },s\right)\psi \left(r,s\right).$$

Thus, in the present case the quantum wave-function $\psi \left(r,s\right)$ depends separately by $r\equiv \left\{r^{\mu }\right\}$ and the proper time *s*, respectively, the center of mass 4-position and proper time associated with its instantaneous 4-position r=r(s), with $\left\{r(s_{1})\right\}\equiv \left\{\left.r(s_{1})\right|r\left(s_{1}\right)\in Q^{4},s_{1}\in I,r\left(s\right)=r\right\}$ being the corresponding space-time trajectory. Here, ψ is a 4-scalar complex function of the form $\psi =\psi \left(r,s\right)$, and $\hat{H}\left(r,\hat{\pi },s\right)$ is a suitable non-local relativistic quantum Hamiltonian operator of the form

(13) $$\hat{H}\left(r,\hat{\pi },s\right)\equiv \frac{1}{2m_{o}c}\left(i\hbar \frac{\partial }{\partial r^{\mu }}+\frac{q}{c}A_{\mu }\left(r,\left[r\right]\right)\right)\left(i\hbar \frac{\partial }{\partial r_{\mu }}+\frac{q}{c}A^{\mu }\left(r,\left[r\right]\right)\right).$$

Notice that here $A_{\mu }\left(r,\left[\bar{r}\right]\right)$ denotes the EM self 4-vector potential containing generally a non-local dependence (represented by the symbol $\left[r\right]$). In fact, by construction this is taken of the form

(14) $$A_{\mu }\left(r,\left[\bar{r}\right]\right)\equiv {\bar{A}}_{\mu }^{\left(ext\right)}\left(r\right)+{\bar{A}}_{\mu }^{\left(self\right)}\left(r,\left[\bar{r}\right]\right),$$

in which ${\bar{A}}_{\mu }^{\left(ext\right)}\left(r\right)$ and ${\bar{A}}_{\mu }^{\left(self\right)}\left(r,\left[\bar{r}\right]\right)$ are, respectively, the contributions due to the external and self EM fields. Then, causal representation of ${\bar{A}}_{\mu }^{\left(self\right)}\left(r,\left[\bar{r}\right]\right)$ can be shown to be

(15) $${\bar{A}}_{\mu }^{\left(self\right)}\left(r,\left[\bar{r}\right]\right)\equiv 2q\int_{-\infty }^{s}ds^{\prime }\frac{dr_{\mu }^{\prime }\left(s^{\prime }\right)}{ds^{\prime }}\delta \left({\tilde{R}}^{\alpha }{\tilde{R}}_{\alpha }-\sigma^{2}\right),$$

where here σ is the invariant radius of the spherical charge distribution, ${\tilde{R}}^{\alpha }\equiv r^{\alpha }\left(s\right)-{\bar{r}}^{\alpha }\left(s^{\prime }\right)$, with *s* and $s^{\prime }$ denoting respectively the present and retarded particle proper times associated with two different causally connected positions occupied by the particle along its trajectory, so that $s^{\prime }<s$ is the positive (causal) root of the delay-time equation ${\tilde{R}}^{\alpha }{\tilde{R}}_{\alpha }-\sigma^{2}=0$. In other words $A_{\mu }\left(r,\left[\bar{r}\right]\right)$ generally depends non-locally on the space-time trajectory $\left\{r(s_{1})\right\}$, the reason being, in fact, that the definite integral on $s^{\prime }$ is effectively a path integral along the causal history of $r^{\mu }\left(s\right)$.

One notices that Equation (12) formally reduces to the Klein–Gordon equation when letting $i\hbar \frac{\partial }{\partial s}\psi \left(r,s\right)=$$k^{2}\psi \left(r,s\right)$ and $\frac{q}{c}A_{\mu }\left(r,\left[r\right]\right)\equiv 0$. However, notable differences of the RR wave Equation (12) (with respect to the Klein–Gordon equation) arise. The first one is that the former is a hyperbolic first-order PDE, the same equation being also non-local due to the characteristic EM RR retarded interaction. Therefore, its solution $\psi \left(r,s\right)$ becomes intrinsically trajectory-dependent, namely, dependent on the (virtual) 4-dimensional quantum trajectory $\left\{r(s_{1})\right\}$ which must associate to each (non-deterministic) 4-position $r\equiv \left\{r^{\mu }\right\}$ the past trajectory followed by the (virtual) relativistic particle which at the present proper time (*s*) crosses the same 4-position *r*. The other distinguishing feature, however, is that $\psi \left(r,s\right)$ is endowed with a strictly positive 4-scalar quantum probability density ${\left|\psi \left(r,s\right))\right|}^{2}\equiv \rho \left(r,s\right)>0$ which holds now on the whole domain of the 4-dimensional Minkowski space-time, rather than on the 3-D Euclidean space Ω. Thus, for two arbitrary functions $\psi_{a}\left(r,t\right)$ and $\psi_{b}\left(r,t\right)$ of such a space their scalar product can again be identified in terms of the *local scalar product* defined as the space-time integral of their product, i.e.,

(16) $$\langle \psi_{a}|\psi_{b}\rangle \equiv \int_{Q^{4}}d\omega \psi_{a}^{*}\left(r,s\right)\psi_{b}\left(r,s\right).$$

Here, dω (in difference with Equation (3)) denotes the 4-dimensional volume element $d\omega =d^{4}r\sqrt{-\left|g_{M}\right|}$ of the Minkowski space-time $Q^{4}$ and $g_{M}$ is the corresponding metric tensor of the same Minkowski space-time. Moreover, for the RR quantum wave equation, however, an alternate prescription analogous to (9) can be conveniently considered for the scalar product. In this case this is achieved letting

(17) $$\langle \langle \psi_{a}|\psi_{b}\rangle \rangle \equiv \frac{\int_{s_{o}}^{s_{o}+\Delta s}ds\int_{Q^{4}}d\omega \psi_{a}^{*}\left(r,s\right)\psi_{b}\left(r,s\right)}{\Delta s}.$$

The remaining notation is as follows: *s* denotes the proper time associated with the particle center of mass, while $\psi_{a}\left(r,s\right)$ and $\psi_{b}\left(r,s\right)$ denote two arbitrary wave functions of the Hilbert space.

The construction of the relevant Heisenberg inequalities becomes then straightforward. Let us determine first the vector inequality extending the position-momentum Heisenberg inequalities (5). For this purpose, let us introduce the conjugate quantum variables represented by the 4-position and its conjugate quantum momentum $\left(r^{\mu },\pi_{\mu }^{\left(q\right)}=-i\hbar \frac{\partial }{\partial r^{\mu }}\right)$, satisfying for μ,ν=0,3 the obvious quantum commutation rule $\left\{\pi_{\mu }^{\left(q\right)},r^{\nu }\right\}=-i\hbar \delta_{\mu }^{\nu }$. Then, the Robertson inequality delivers (in analogy to Equation (5)) the 4-vector inequality

(18) $$\sigma_{r^{\left(\mu \right)}}\sigma_{\pi_{\left(\mu \right)}^{\left(q\right)}}\geq \frac{\hbar }{2},$$

namely holding for the whole set of indexes μ=0,1,2,3, with $\sigma_{r^{\mu }}$ and $\sigma_{\pi_{\mu }^{\left(q\right)}}$ denoting the 4-position and corresponding quantum 4-momentum standard deviations, with $\sigma_{r^{\mu }}^{2}$ and $\sigma_{\pi_{\mu }^{\left(q\right)}}^{2}$ being the corresponding variances. Notice that here the local scalar product (16) has been used, so that the standard deviations are defined accordingly, namely, letting as in [2], $\sigma_{r^{\mu }}^{2}=\langle \psi \left|{\left(r^{\mu }-{\bar{r}}^{\mu }\right)}^{2}\psi \right.\rangle$ and $\sigma_{\pi_{\mu }^{\left(q\right)}}^{2}=\langle \psi \left|{\left(\pi_{\mu }^{\left(q\right)}-\bar{\pi_{\mu }^{\left(q\right)}}\right)}^{2}\psi \right.\rangle$.

In terms of the conjugate 4-scalar canonical variables $\left(s,\pi_{s}^{\left(q\right)}=-i\hbar \frac{\partial }{\partial s}\right)$ one obtains instead the single (i.e., 4-scalar) inequality

(19) $$\sigma_{s}\sigma_{\pi_{s}^{\left(q\right)}}\geq \frac{\hbar }{2},$$

where now the standard deviations are defined with respect to the non-local scalar product (17), so that $\sigma_{s}^{2}=\langle \langle \psi \left|{\left(s-\bar{s}\right)}^{2}\psi \right.\rangle \rangle$ and $\sigma_{\pi_{s}^{\left(q\right)}}^{2}=\langle \langle \psi \left|{\left(\pi_{s}^{\left(q\right)}-\bar{\pi_{s}^{\left(q\right)}}\right)}^{2}\psi \right.\rangle \rangle$. Therefore, in the framework of the RQM for an extended charged spinless particle subject to the action of the electromagnetic (EM) radiation-reaction (RR) interaction, inequalities (18) and (19) represent, respectively, the *position-momentum* and *proper-time-extended canonical momentum Heisenberg inequalities.*

### 2.3. Conditions of Validity and Interpretation

A peculiar feature of the Heisenberg inequalities reported above (see Equations (5) and (18), and, respectively, (11) and (19)) should be stressed. This is that the same inequalities *do not hold for arbitrary wave functions* ψ. The exception in fact happens respectively when ψ is an eigenfunction of the corresponding canonical momentum operator, i.e., for the 3-vector and scalar operators $\pi_{r^{i}}^{\left(q\right)}$ and $\pi_{t}^{\left(q\right)}$ in NRQM and for the 4-vector and scalar operators $\pi_{r^{\mu }}^{\left(q\right)}$ and $\pi_{s}^{\left(q\right)}$. Thus, for example, in case of energy eigenstates for which $\psi \left(r,t\right)=\hat{\psi }\left(r\right)e^{-\frac{i}{\hbar }Et}$ one has in fact

(20) $$p_{t}\psi =-E\psi \left(r,t\right)=-H\psi ,$$

with *E* being the energy eigenvalue. This assumption, however, implies $\bar{p_{t}}=\langle \psi \left|p_{t}\psi \right.\rangle =-E$ so that $\sigma_{p_{t}}^{2}=\langle \psi \left|{\left(p_{t}-\bar{p_{t}}\right)}^{2}\psi \right.\rangle =\langle \psi \left|\left(E^{2}+2Ep_{t}+E^{2}\right)\psi \right.\rangle =0$, which means that the second inequality (11) is manifestly violated. The analogous conclusion applies of course in the case ψ(r,t) is an eigenfunction of the vector canonical momentum operator $p_{r^{i}}$, as mentioned above.

The interpretation of the two sets of inequalities is, apart certain subtleties, substantially coherent with the customary one available in QM.

Let us consider first the position-conjugate momentum inequalities in the two cases discussed above. The first one (5) (and similarly (18)) represents a constraint arising on the simultaneous quantum measurements of the *i*-th Lagrangian coordinate $r^{i}$ and its canonically-conjugate momentum operator $p_{r^{i}}$.Here, i=1,2,3 is a tensor index, so that the corresponding inequality has a 3-vector character. The relativistic inequality (18) reveals important features of RQM involved in Equation (12). The first one is that the position-canonical momentum inequality (18) is actually a 4-vector inequality. Thus, in particular its time component (μ=0) prescribes the uncertainty occurring on the simultaneous measurement of the coordinate time and the corresponding conjugate momentum. The second feature to be remarked is that, contrary to the customary assumption introduced in the Klein–Gordon equation, ${\left|\psi \left(r,s\right)\right|}^{2}\equiv \rho \left(r,s\right)>0$ in Equation (12) is by construction a probability density on the whole Minkowski space-time, i.e., such that identically $\langle \psi |\psi \rangle =\langle \langle \psi |\psi \rangle \rangle =1$. Therefore, it is necessarily a 4-scalar (and not the time component of a 4-tensor as in the customary treatment of the Klein–Gordon equation).

Regarding the scalar inequalities (11) and (19), although formally similar, their physical interpretation is different. In fact the non-relativistic inequality (11) should be rather compared with the time (i.e., μ=0) component of 4-vector inequality (18) which actually yields a similar bound from below for simultaneous measurement of the coordinate time variable and the extended conjugate canonical momentum operator. The inequality (19) is peculiar in that it introduces the quantum proper time *s* and its conjugate momentum operator $\pi_{s}^{\left(q\right)}$. This is possible because the quantum wave function ψ(r,s) is, by construction, dependent on the past space-time trajectory $\left\{r(s_{1})\right\}$. Thus, the meaning of inequality (19) is related to the product of the “uncertainties” (represented in terms of standard deviations) associated with the quantum proper time *s* and its conjugate momentum, both associated with a particle traveling along its space-time trajectory. As both quantities are 4-scalars it is obvious that the inequality (19) is unrelated to the spatial component of inequality (18).

As a consequence, the uncertainties arising in the product of two conjugate physical observables is in all cases necessarily bounded from below by the corresponding Heisenberg inequality, namely either (5), (11) and, respectively, (18) and (19). These inequalities, however, hold true only provided the quantum state is not an eigenstate of the involved quantum operators. In fact, if this happens the corresponding quantum observable is known deterministically and hence no possible uncertainty in its measurement can arise. Quantum states of this type, i.e., which are not eigenfunctions of the stated operators, can easily be found in the literature. An example of non-eigenfunction solution holding in the case of NRQM for which both Heisenberg inequalities (5) and (11) hold true is the Gaussian solution determined in [7] and based on the trajectory-based generalized Lagrangian path (GLP) approach to NRQM [21] (see also in [22] for a discussion about HIP).

## 3. Setting the Problem in the Context of Quantum Gravity

By a simple criterion of analogy one expects that also in the context of quantum field theory, in particular for QG, analogous Heisenberg inequalities should apply for the corresponding set of (continuous) canonical field variables and operators. In particular, this means that in the framework of a manifestly-covariant treatment, all these quantities, in particular the continuous Lagrangian coordinates and corresponding operators should be endowed with tensor properties with respect to a suitable group of coordinate transformations. To be clear on this crucial point, however, such a group cannot be naively identified with the Lorentz (or Poincaré) group, but with the full group of local point transformations

(21) $$r\to r^{\prime }\left(r\right)$$

(LPT-group) which leaves invariant the differential manifold of a suitable background curved space-time, to be prescribed below. This is, indeed, the proper meaning of manifest covariance. This means, in the present context, that it should be possible to identify 4-tensor conjugate canonical variables analogous to the 3-vector set (r,p), as well as appropriate corresponding 4-scalar proper-time and Hamiltonian operator variables analogous to the set (t,H) represented by coordinate time and non-relativistic Hamiltonian operator. As a consequence, one expects also for QG the validity of corresponding *conjugate-canonical variables Heisenberg inequalities*, relating the product of the standard deviations of the relevant canonically-conjugated quantum canonical variables, and a *proper-time-extended canonical momentum inequality,* relating the product of the standard deviation of the relevant 4-scalar proper length (or proper time) and the quantum Hamiltonian operator.

The precise reason why in the present context manifest covariance is so crucial is that QG-theory should be necessarily such to permit a second-quantization theory for the gravitational field, in the sense of yielding also the non-linear quantum modifications of the background space-time. Thus, QG-theory should predict also the related quantum-modified form of Einstein Field Equations (EFE) which determines the background metric field tensor. As a consequence, the same equation—just as the original EFE—should be necessarily realized by means of a tensor (and therefore frame-independent) PDE, so that it must preserve its form with respect to the group of local point coordinate transformations between two arbitrary GR-frames. The implication is therefore that QG-theory should have a frame- (i.e., coordinate-) independent character so to result intrinsically manifestly-covariant in form. This should therefore be regarded as a mandatory property ultimately stemming from the standard formulation of GR itself.

Let us briefly analyze some possible mandatory requirements which are needed in order to satisfy HIP in the context of QG theory. The first one is that the same Heisenberg inequalities, to make sense at all in the context of GR and covariant QG, should be identified with well-defined tensor fields, so that this means that they should satisfy the property of manifest covariance. As such, they should transform according to the 4-tensor transformation laws determined by the group of local point transformations (LPT-group), i.e., diffeomorphisms of the form (21), between different coordinate systems (GR-frames) which map in itself the space-time. For definiteness the latter is being identified with the 4-dimensional Lorentzian differential manifold $\left\{Q^{4},\hat{g}\left(r\right)\right\}$ with signature $\left(+,-,-,-\right)$ (or analogue permutations). Here, $r=\left\{r^{\mu }\right\}$ and $r^{\prime }=\left\{r^{\prime }{}^{\mu }\right\}$ denote two arbitrary 4-positions (or coordinate systems) belonging to the same “background” space-time structure. Moreover, $Q^{4}$ denotes a 4-dimensional Riemann space-time with suitably-prescribed symmetric “background” metric tensor $\hat{g}\left(r\right)\equiv \left\{{\hat{g}}_{\mu \nu }\left(r\right)\right\}$ and Riemannian length

(22) $$ds^{2}={\hat{g}}_{\mu \nu }\left(r\right)dr^{\mu }dr^{\nu }.$$

Thus, it means that the Heisenberg inequalities should be independent of the choice of the coordinate system, so that their prescription is “frame-independent”.

The second requirement concerns the mandatory tensor property of QG which emerges by the nature itself of the Heisenberg inequalities. In fact, these should be related to quantum expectation values of a complex quantum wave function ψ belonging to a Hilbert space. In particular, in the context of QG the following *physical requirements* should hold.

- *(Phys.Req.#1*) Both ψ and ${\left|\psi \right|}^{2}$ should be 4-scalars.
- *(Phys.Req.#2)* In case of validity of the quantum unitarity principle and validity of the Born rule, ${\left|\psi \right|}^{2}$ should be a probability density.
- *(Phys.Req.#3)* In order to be able to recover the proper-time-extended canonical momentum inequality indicated above, it goes without saying that the quantum wave function should depend also on the same proper time (*s*), to be suitably prescribed and associated with the background Lorentzian differential manifold $\left\{Q^{4},\hat{g}\left(r\right)\right\}$.
- *(Phys.Req.#4)* All expectation values of quantum observables actually identify classical fields of GR. This means that both the quantum observables and their corresponding quantum expectation values must necessarily be identified with 4-tensors with respect to the LPT-group (21) acting on the background space-time $\left\{Q^{4},\hat{g}\left(r\right)\right\}$.
- *(Phys.Req.#5)* Both in validity of quantum unitarity or not, the quantum wave function ψ should span a Hilbert space. This means that in both cases it should be possible to define a scalar product on a suitable functional linear space.

The question which then emerges is therefore the identification of the appropriate quantum theory of gravity that fulfills these criteria. It is easy to prove, nevertheless, that such a program fails in the case of the quantum-wave equation which is at the basis of some of the most popular QG theories [23,24], namely the Wheeler–DeWitt equation [25] which prescribes the (stationary) quantum wave-function Ψ(r). In fact, it is well-known that its solution, namely the wave function Ψ(r), known as “*wave function of the universe*” [26], is not a 4-scalar. In addition, despite its formal analogy with the Schroedinger equation, crucial differences and inconsistencies emerge with the requirements indicated above.

The first one is that the same equation, and consequently also Ψ(r) itself, is intrinsically frame-dependent, namely, it violates explicitly the property of manifest covariance. This occurs because of the explicit parametrization adopted in its derivation which is based on the coordinate-time *t*, and not on an invariant 4-scalar parameter. The second reason is that also ${\left|\Psi (r)\right|}^{2}$ is not a 4-scalar. This implies therefore that in accordance with the Born rule ${\left|\Psi (r)\right|}^{2}$ cannot realize a probability density. As a consequence, both the principle of quantum unitarity and the Born rule do not hold for the Wheeler–DeWitt equation, while the customary notions of scalar product and Hilbert space do not apply either. Therefore, the Wheeler–DeWitt equation cannot admit, in a proper sense, Heisenberg inequalities which make sense from the physical standpoint, such as the analog conjugate-canonical variables and time-extended momentum Heisenberg inequalities. Finally, it is obvious that, for the same reason, functionals of ${\left|\Psi (r)\right|}^{2}$ cannot trivially prescribe 4-tensors. The consequence is that even if one might construct some new kind of fancy inequalities (possibly analogous in some sense to the Heisenberg ones) they would never possibly exhibit the correct 4-tensor properties.

However, as earlier pointed out [27], the physical origin of the failure of the Wheeler–DeWitt equation lies in the non-relativistic nature of the same equation. The latter, in fact, is based on a preliminary 3+1 foliation of space-time which intrinsically violates the principle of general covariance, i.e., the coordinate-independent property characteristic of GR, according to which the foliation should be preserved by the whole LPT-group (21). This feature is inherited by the quantum Wheeler-DeWitt theory from the well-known 3+1 Arnowitt–Deser–Misner (ADM) Hamiltonian formulation underlying classical GR [28]. The non-relativistic character of the ADM representation is nevertheless obvious, as it involves the prescription of a suitable family of GR-frames which are connected only by a subset of point transformations (21) which do not mix time and space coordinates. Such a feature rules out by itself, already at the classical level, the property of manifest covariance of the theory. This supports the objection raised by Hawking against the ADM theory, who stated that “the split into three spatial dimensions and one time dimension seems to be contrary to the whole spirit of Relativity” [29]. However, it must be stressed that, despite this characteristic, the adoption of non-covariant approaches to classical GR and quantum gravity is widespread and can be helpful in cases where the choice of a “preferred frame” may exist. Examples include numerical relativity [30], classical and quantum cosmology [31,32,33,34,35], quantum-field phenomena associated with black-hole physics [36,37].

The previous considerations unavoidably leave us with the task of seeking a possible alternative theoretical approach to quantum gravity. *It seems obvious, in fact, that the relativistic generalization of the Wheeler–DeWitt equation should be achieved by means of a suitable manifestly-covariant equation.* In this regard, as the property of manifest covariance of a theory is associated with its representation, it follows that Phys.Req.#1 and Phys.Req.#3 provide necessary conditions for the formalism to be manifestly covariant. As we intend to show below this task is realized by the theory of manifestly-covariant quantum gravity (CQG-theory) and the related manifestly-covariant quantum wave equation, earlier developed in [5,6]. Its construction is based on the discovery of classical and corresponding non-perturbative quantum Hamiltonian structures of GR which are mutually related by means of standard covariant canonical quantization methods.

## 4. Realizations of CQG Theory and Corresponding Hilbert-Space Setting

In this section, we consider the construction of a theory of QG, and a corresponding quantum-wave equation, both of which satisfy the physical requirements indicated above, i.e., *Phys.Req.#1-#5*. The first main implication is the validity of the principle of manifest covariance. This is a prerequisite in order to be able to identify all physical observables in terms of 4-tensors (*Phys.Req.#4*). In fact, in order to warrant that the quantum wave function ψ(s) and its square modulus ${\left|\psi \right|}^{2}$ remain 4-scalars for all values of *s* and for arbitrary choices of the GR-frame (*Phys.Req.#1*), their realization should be coordinate-independent. In other words, the quantum wave equation itself must be manifestly-covariant too, namely expressed in 4-tensor form. The second implication concerns the physical role of ${\left|\psi \right|}^{2}$. In validity of quantum unitarity this should coincide with a quantum probability density (*Phys.Req.#2*). As a consequence, ${\left|\psi \right|}^{2}$ should therefore satisfy an appropriate manifestly-covariant quantum continuity equation. It is obvious, however, that the latter equation cannot be independent of the aforementioned quantum wave equation (otherwise it would place an extra non-trivial constraint on the same wave-function ψ(s)). The third implication is that, in order to warrant at least local existence for the solution of the quantum wave equation, the latter should be realized by means of an evolution (i.e., hyperbolic) equation with respect to an appropriate time variable *s*, while the same variable should be necessarily a 4-scalar. A parameter of this kind consistent with the principle of manifest covariance is the Riemann distance prescribed along field geodetics $\left\{\left.r(s)\right|s\in \left[s_{0},s_{1}\right]\right\}$ of the same background space-time $\left(Q^{4},\hat{g}\left(r\right)\right)$. From such premises, although the choice of theoretical framework remains still intrinsically non-unique, it follows that a candidate theory of QG which possesses all the required characteristics is the theory of manifestly-covariant quantum gravity (CQG-theory). The corresponding quantum wave equation, denoted as CQG-wave equation, takes the form of a hyperbolic evolution equation (see in [2,6])

(23) $$i\hbar \frac{\partial \psi (s)}{\partial s}=\left[H^{\left(q\right)},\psi \left(s\right)\right]\equiv H^{\left(q\right)}\psi \left(s\right),$$

subject to an initial condition of the type

(24) $$\psi \left(s_{0}\right)=\psi_{0}\left(g,\hat{g}\left(r\left(s_{0}\right),s_{0}\right),r\left(s_{0}\right)\right),$$

with $\psi_{0}\left(g,\hat{g}\left(r\left(s_{0}\right),s_{o}\right),r\left(s_{0}\right)\right)$ denoting an appropriate and smooth initial wave function and ∂/∂s the covariant s-derivative. Here, $H^{\left(q\right)}$ is a prescribed quantum Hamiltonian operator (see Appendix A and discussion below), while ψ(s)≡$\psi (g,\hat{g},r,s)$ with $\hat{g}\equiv \hat{g}\left(r\left(s\right),s\right)$, is a smooth complex quantum wave function associated with a massive graviton and defined on the ten-dimensional configuration space $U_{g}\subseteq R^{10}$ which is spanned by the real symmetric tensor field $g\equiv \left\{g_{\mu \nu }\right\}$. Here, $\hat{g}\equiv \left\{{\hat{g}}_{\mu \nu }\right\}$ denotes the background field tensor associated with $\left(Q^{4},\hat{g}\left(r\right)\right)$, which can be assumed of the general form ${\hat{g}}_{\mu \nu }\equiv {\hat{g}}_{\mu \nu }\left(r,s\right)$ [8]. Furthermore, r=r(s) is the 4-position along a local field geodetics $\left\{\left.r(s)\right|s\in \left[s_{0},s_{1}\right]\right\}$ spanning the background space-time $\left(Q^{4},\hat{g}\left(r,s\right)\right)$ and *s* is the Riemann length evaluated along a suitable family of field geodetics belonging to *r* and assumed to be the same for all such curves.

Despite its apparent simplicity, Equation (23) is of paramount importance. In fact, as discussed elsewhere in detail [7,8,12], besides the quantum wave function ψ the same equation actually prescribes uniquely also the evolution of the background metric tensor $\hat{g}\equiv \left\{{\hat{g}}_{\mu \nu }\left(r,s\right)\right\}$ which determines the geometry of space-time.

### 4.1. The Unitary Realization of CQG-Theory

In order that Equation (23) makes sense, the Hamiltonian operator $H^{\left(q\right)}$ and the related Hilbert space spanned by ψ(s) must also be prescribed. Thus, in particular, in order to satisfy the Hilbert-space requirement (*Phys.Req.#5*), a suitable definition of scalar product $\langle \langle \psi_{a}\left(s\right)|\psi_{b}\left(s\right)\rangle \rangle$ must be given. This prescription is needed to warrant the validity of the principle of quantum unitarity. The unitary representation of CQG-theory is based on the requirement that

(25) $$H^{\left(q\right)}\equiv H_{R}^{\left(q\right)},$$

where $H_{R}^{\left(q\right)}$ is recalled in Appendix B (see Equation (A1)). However, quantum unitarity, i.e., validity for arbitrary s∈I≡R of the normalization constraint

(26) $$\langle \langle \psi (s)|\psi (s)\rangle \rangle =1,$$

requires $H_{R}^{\left(q\right)}$ to be a Hermitian operator, i.e., to satisfy, in terms of the sought scalar product, also the identity

(27) $$\langle \langle \psi \left(s\right)\left|H_{R}^{\left(q\right)}\psi \left(s\right)\right.\rangle \rangle =\langle \langle \left.H_{R}^{\left(q\right)}\psi \left(s\right)\right|\psi \left(s\right)\rangle \rangle .$$

In analogy to the definition holding in QM (see Equation (9)), a convenient alternative prescription of the scalar product suitable for the task can be introduced as follows,

(28) $$\langle \langle \psi_{a}|\psi_{b}\rangle \rangle \equiv \frac{1}{\int_{s_{0}}^{s_{1}}ds}\int_{s_{0}}^{s_{1}}ds{\langle \psi_{a}|\psi_{b}\rangle }_{L},$$

where a proper-time integration on the interval $\left[s_{0},s_{1}\right]$ (with $s_{0},s_{1}\in I\subseteq R$) has been included for later convenience. Here, ${\langle \psi_{a}|\psi_{b}\rangle }_{L}$ denotes the customary definition, i.e., the *local* scalar product

(29) $${\langle \psi_{a}|\psi_{b}\rangle }_{L}\equiv \int_{U_{g}}d\left(g\right)\psi_{a}^{*}\left(g,\hat{g}\left(r,s\right),r\left(s\right),s\right)\psi_{b}\left(g,\hat{g}\left(r,s\right),r\left(s\right),s\right),$$

where $\psi_{a}\equiv \psi_{a}\left(g,\hat{g}\left(r,s\right),r\left(s\right),s\right)$ and $\psi_{b}\equiv \psi_{b}\left(g,\hat{g}\left(r,s\right),r\left(s\right),s\right)$ are two arbitrary elements of the Hilbert space. We notice that ${\langle \psi_{a}|\psi_{b}\rangle }_{L}$ is still a function of the arguments $\hat{g}\left(r\right)$, r(s) and *s*. Thus, while generally for an arbitrary Hermitian quantum operator *A* one has that $\langle \langle \psi_{a}|A\psi_{b}\rangle \rangle \neq {\langle \psi_{a}|A\psi_{b}\rangle }_{L}$, thanks to Equation (26) by construction it follows,

(30) $$\langle \langle \psi (s)|\psi (s)\rangle \rangle \equiv \int_{U_{g}}d\left(g\right){\left|\psi (g,\hat{g}\left(r,s\right),r\left(s\right),s)\right|}^{2}\equiv \langle \psi (s)|{\psi (s)\rangle }_{L}.$$

This equation has been obtained setting $\psi_{a}^{*}\equiv \psi^{*}\left(s\right)$, $\psi_{b}\equiv \psi \left(s\right)$, with $\psi \left(s\right)\equiv \psi (g,\hat{g}\left(r,s\right),r\left(s\right),s)$ denoting and arbitrary element of the same Hilbert space, such as a particular solution of the CQG-wave Equation (23). Notice furthermore that here the equality of the rhs being warranted by the fact that in the unitary case $\langle \psi (s)|{\psi (s)\rangle }_{L}$ is independent of proper time *s*.

We stress that the CQG-quantum wave Equation (23) holds in principle for arbitrary suitably-smooth initial conditions set at an (arbitrary) initial proper time, as well as in principle arbitrary external sources and arbitrary boundary conditions prescribed on smooth hypersurfaces, such as event horizons. The implication is important because it means that it should be possible to use the freedom in the choice of the initial condition for $\psi (s_{0})$ in order not only to recover the Einstein field equations (EFE) but to identify also the possible relevant second-quantization corrections.

This line of investigation has been pursued in [7,8] and further on in [27,38]. Thus, by suitably prescribing the initial quantum PDF $\rho \left(s_{0}\right)\equiv {\left|\psi (s_{0})\right|}^{2}$ as well as the complex part of $\psi (s_{0})$, together with appropriate boundary conditions in vacuum, the so-called *emergent-gravity property* characteristic of CQG-theory was uncovered. This arises because in such a case Equation (23) can be shown to determine the classical background space-time metric tensor $\hat{g}\left(r\right)$. Indeed, the same field tensor is actually found to coincide with the quantum expectation value of an underlying quantum field [7] and, for appropriate initial conditions, the same Equation (23) recovers a representation formally analogous to the Einstein field equation (EFE), including the possible quantum prescription of the cosmological constant (CC). In particular, in this way a number of new notable theoretical/physical models have been advanced which explain the following.

- The quantum origin of the CC, which arises in fact solely due to second-quantization effects, i.e., due to the Bohm vacuum interaction acting among, otherwise free, gravitons [8].
- The possible existence of a quantum screening effect of the quantum CC affecting its absolute value [27].
- The physical interpretation of the classical CC and its relationship with the corresponding quantum CC [38].

### 4.2. Prescription of Proper Time *s* and Field Geodetics

The construction of the Covariant Quantum Gravity in terms of CQG-theory involves the prescription of the proper time *s* as well as of the (non-null) field geodetics, each one characterized by its local tangent vector field (say *n*). The basic assumptions are that:

(1) *Assumption #1:* Let us denote by $\left\{Q^{\left(4\right)},\hat{g}\right\}$ the differential manifold spanned by the considered field geodetics $\left\{\left.r(s)\right|s\in \left[s_{0},s_{1}\right]\right\}$ and associated with the background metric field tensor determined by CQG-theory. Thus, $\hat{g}\left(r,s\right)$ in a given GR-frame takes the form $\hat{g}\equiv \left\{{\hat{g}}_{\mu \nu }\left(r,s\right)\right\}$, with $r\equiv \left\{r^{\mu }\right\}$ denoting the generic 4-position. Here, *s* is the proper time and ds its arc-length element along suitably-defined field geodetics, with $ds^{2}={\hat{g}}_{\mu \nu }\left(r,s\right)dr^{\mu }dr^{\nu }$.

(2) *Assumption #2:* In order to prescribe uniquely *s* it is assumed that there exists a single s=0 hypersurface Σ(s=0), with normal n(s=0), which belongs to the background differential manifold $\left\{Q^{\left(4\right)},\hat{g}\right\}$ from where all of the considered field non-null geodetics belong. In addition, depending on the choice of the s=0 hypersurface (which remains in principle arbitrary and dependent in turn on the prescription of the background metric tensor field $\hat{g}\left(r,s\right)$), on the same hypersurface Σ(s=0) all tangent vectors *n* are required to satisfy suitable initial conditions n(s=0). Here, the notation is as follows; $\hat{g}\left(r,s\right)$ is the background metric field tensor which in a given GR-frame takes the form $\hat{g}\equiv \left\{{\hat{g}}_{\mu \nu }\left(r,s\right)\right\}$, $r\equiv \left\{r^{\mu }\right\}$ is the 4-position, *s* is the proper time evaluated along each non-null field geodetics in terms of ds, the arc-length along the same geodetics, namely, such that $ds^{2}={\hat{g}}_{\mu \nu }\left(r,s\right)dr^{\mu }dr^{\nu }$.

(3) *Assumption #3:* In a given arbitrary GR-frame, at each 4-position $r\equiv \left\{r^{\mu }\right\}$, there is a unique field geodetics $\left\{\left.r(s)\right|s\in \left[s_{0},s_{1}\right]\right\}$ belonging to it which corresponds to a prescribed choice of the s=0 hypersurface.

(4) *Assumption #4:* The prescription of the background metric tensor field $\hat{g}\left(r,s\right)$ remains nevertheless arbitrary.

The set of these assumptions can be viewed as the proper GR generalization of the non-relativistic framework underlying the 3+1 decomposition of space-time in the Wheeler–DeWitt theory. In fact, Assumptions #1-#4 allow one to construct a family hypersurfaces of space-time characterized by the invariant proper-time parameter *s* and satisfying in this way the property of manifest covariance. As a consequence, this formalism warrants that CQG-theory is invariant with respect to a change of background metric tensor field $\hat{g}\left(r,s\right)$, with the s=0 hypersurface Σ(s=0) being uniquely associated to that.

## 5. Proof of the Heisenberg Indeterminacy Principle in CQG-Theory

In this section the main goal of the investigation is presented, which concerns the first-principle proof of the Heisenberg indeterminacy principle for the CQG-wave Equation (23). More precisely, the problem is addressed whether, as a consequence of the strict positivity and smoothness of the quantum PDF ρ(s) and in analogy with standard quantum mechanics, the quantum state ψ(s) might/should satisfy suitable generalized Heisenberg inequalities which are related to the fluctuations (and corresponding standard deviations) of the Lagrangian variables and of conjugate quantum canonical momenta, and if the same inequalities might place a constraint on the proper-time evolution of the quantum state.

Denoting for brevity $\psi_{a}=\psi_{a}\left(s\right)$ and $\psi_{b}=\psi_{b}\left(s\right)$, we notice that the definitions of scalar product introduced in Section 4 satisfy the standard properties of the Cauchy–Schwartz inequality. Thus, by construction

(31) $$\langle \langle \psi_{a}|\psi_{a}\rangle \rangle \langle \langle \psi_{b}|\psi_{b}\rangle \rangle \geq {\left|\langle \langle \psi_{a}|\psi_{b}\rangle \rangle \right|}^{2}.$$

On the other hand it is easily proved that

(32) $$\left|{\left(\langle \langle \psi_{a}|\psi_{b}\rangle \rangle \right)}^{2}\right|\geq {\left|\frac{1}{2i}\left(\langle \langle \psi_{a}|\psi_{b}\rangle \rangle -\langle \langle \psi_{b}|\psi_{a}\rangle \rangle \right)\right|}^{2},$$

and we notice that by construction

(33) $$\langle \langle \psi_{a}|\psi_{b}\rangle \rangle ={\langle \langle \psi_{b}|\psi_{a}\rangle \rangle }^{*}.$$

Now, consider two Hermitian operators *A* and *B*, denoting

(34) $$\begin{array}{ccc} |\psi_{a}\rangle \rangle & = & |(A-a)\psi \rangle \rangle , \end{array}$$

(35) $$\begin{array}{ccc} |\psi_{b}\rangle \rangle & = & |(B-b)\psi \rangle \rangle , \end{array}$$

with *a* and *b* being the expectation values $\langle \langle \psi |A\psi \rangle \rangle =a$ and $\langle \langle \psi |B\psi \rangle \rangle =b$. In addition, let us assume for definiteness that the quantum wave function ψ is not an eigenfunction of either operators, which would require identically either

(36) (A−a)ψ≡0,

or

(37) (B−b)ψ≡0.

Thus, excluding such occurrences it follows that

(38) $$\begin{array}{ccc} \langle \langle \psi_{a}|\psi_{a}\rangle \rangle & = & \sigma_{A}^{2}\equiv \langle \psi |{\left(A-a\right)}^{2}\psi \rangle >0, \end{array}$$

(39) $$\begin{array}{ccc} \langle \langle \psi_{b}|\psi_{b}\rangle \rangle & = & \sigma_{B}^{2}\equiv \langle \psi |{\left(B-b\right)}^{2}\psi \rangle >0, \end{array}$$

where $\sigma_{A}$ and $\sigma_{B}$ denote the standard deviations of the quantum operators *A* and *B*. Furthermore, one can then show that under the same assumption of Hermiticity of the same operators *A* and *B* it follows that

(40) $$\langle \langle \psi_{a}|\psi_{b}\rangle \rangle -\langle \langle \psi_{b}|\psi_{a}\rangle \rangle ={\left|\frac{1}{2i}\langle \langle \psi |\left[A,B\right]\psi \rangle \rangle \right|}^{2}.$$

We conclude therefore that also in the context of CQG-theory the formal inequality

(41) $$\sigma_{A}^{2}\sigma_{B}^{2}\geq {\left|\frac{1}{2i}\langle \langle \psi |\left[A,B\right]\psi \rangle \rangle \right|}^{2},$$

holds, namely

(42) $$\sigma_{A}\sigma_{B}\geq \left|\frac{1}{2i}\langle \langle \psi |\left[A,B\right]\psi \rangle \rangle \right|,$$

which is usually referred to as *Robertson uncertainty relation, and actually expresses the Heisenberg indeterminacy principle*. For completeness, we stress that analogous inequalities can be determined in terms of the local scalar product, thus yielding

(43) $$\sigma_{A}\sigma_{B}\geq \left|\frac{1}{2i}\langle \psi |\left[A,B\right]\psi \rangle \right|,$$

where the standard deviations are again prescribed in terms of the local scalar product defined above. The appropriate choice of the inequalities depends, as in the case of the QM treatment considered in Section 2.1, on the Hermitian properties of the operators *A* and *B* (i.e., whether they are fulfilled or not by the local scalar product, rather than by the non-local one). In both cases the meaning of the inequalities is nevertheless analogous. More precisely, they state that the product of the standard deviations of two—in principle arbitrary—non-commuting quantum observables, *A* and *B*, is bounded from below by one half of the modulus of the expectation value of the commutator $\left[A,B\right]$. The result yields also the conditions of validity of the indeterminacy principle in the context of QG, which are analogous to those applying in QM. In other words, HIP holds only provided the quantum wave-function ψ is not an eigenfunction of either operators.

However, what appears significant is that HIP exhibits a universal character. In fact, it holds also for the realization of the scalar product valid in the case considered above, i.e., identified according to the prescription (28).

## 6. Heisenberg Inequalities: Case of Effectively Conjugate Variables

We now consider specifically the choices of the operators *A* and *B* in which they coincide with canonically-conjugate quantum variables or are related to them. Three possible cases are analyzed in detail.

### 6.1. Conjugate-Canonical Variables Heisenberg Inequalities

The first type of inequalities corresponds to the obvious identification in terms of the conjugate quantum canonical variables:

(44) $$\left(A,B\right)=\left(g_{\mu \nu },\pi_{\mu \nu }^{\left(q\right)}\equiv -i\hbar \frac{\partial }{\partial g^{\mu \nu }}\right),$$

for which the commutator is obviously

(45) $$\left[g_{\left(\mu )(\nu \right)},\pi_{\mu \nu }^{\left(q\right)}\right]=i\hbar .$$

It therefore follows the inequality for the corresponding standard deviations

(46) $$\sigma_{g_{\left(\mu )(\nu \right)}}\sigma_{\pi_{\left(\mu )(\nu \right)}^{\left(q\right)}}\geq \frac{\hbar }{2},$$

which realizes the (generalized) Heisenberg inequality for this set of conjugate canonical variables (44). The corresponding fluctuations (squared standard deviations) are given in terms of the local scalar product by

(47) $$\begin{array}{ccc} \sigma_{g_{\mu \nu }}^{2} & \equiv & \langle {\left(g_{\left(\mu )(\nu \right)}-{\tilde{g}}_{\left(\mu )(\nu \right)}\right)}^{2}\rangle \equiv \langle \langle \psi |{\left(g_{\left(\mu )(\nu \right)}-{\tilde{g}}_{\left(\mu )(\nu \right)}\right)}^{2}\psi \rangle \rangle , \end{array}$$

(48) $$\begin{array}{ccc} \sigma_{\pi_{\mu \nu }^{\left(q\right)}}^{2} & \equiv & \langle {\left(\Delta \pi_{\mu \nu }^{\left(q\right)}\right)}^{2}\rangle \equiv \langle \langle \psi |{\left(\Delta \pi_{\mu \nu }^{\left(q\right)}\right)}^{2}\psi \rangle \rangle , \end{array}$$

with the short-way notation $\Delta \pi_{\mu \nu }^{\left(q\right)}=\pi_{\left(\mu )(\nu \right)}^{\left(q\right)}-{\tilde{\pi }}_{\left(\mu )(\nu \right)}^{\left(q\right)}$, where ${\tilde{g}}_{\left(\mu )(\nu \right)}$ and ${\tilde{\pi }}_{\left(\mu )(\nu \right)}^{\left(q\right)}$ are the quantum expectation values.

We remark that the generalized Heisenberg inequality (46) is analogous to that earlier reported in [7] for the wave function ψ(s) and expressed in terms of the local scalar product (29). The present inequality (46) applies to the case of a non-stochastic wave-function ψ(s).

Another important remark concerns the evaluation of the momentum fluctuation $\langle {\left(\Delta \pi_{\mu \nu }^{\left(q\right)}\right)}^{2}\rangle$. In analogy to the work in [7] one can show that also in the present case it can be decomposed in the form

(49) $$\langle {\left(\Delta \pi_{\mu \nu }^{\left(q\right)}\right)}^{2}\rangle ={\langle {\left(\Delta \pi_{{}_{\mu \nu }}\right)}^{2}\rangle }_{1}+{\langle {\left(\Delta \pi_{{}_{\mu \nu }}\right)}^{2}\rangle }_{2},$$

with ${\langle {\left(\Delta \pi_{{}_{\mu \nu }}\right)}^{2}\rangle }_{1}$ and ${\langle {\left(\Delta \pi_{{}_{\mu \nu }}\right)}^{2}\rangle }_{2}$ denoting, respectively, the weighted integrals:

(50)Δπμν21=ℏ24F∫Ugd(g)ρ∂lnρ∂gμν∂lnρ∂g(μ)(ν),(51)Δπμν22=F∫Ugd(g)ρ∂S(q)∂gμν−π˜μν∂S(q)∂g(μ)(ν)−π˜(μ)(ν),

where *F* is the linear operator $F=\frac{1}{\Delta s}\int_{s_{0}}^{s_{0}+\Delta s}ds$. One can then show (see details in [7]) that the relation (46) can actually be replaced by the stronger fluctuation inequality

(52) $$\langle {\left(g_{\left(\mu )(\nu \right)}-{\tilde{g}}_{\left(\mu )(\nu \right)}\right)}^{2}\rangle {\langle {\left(\Delta \pi_{\mu \nu }\right)}^{2}\rangle }_{1}\geq \frac{\hbar^{2}}{4},$$

and therefore also that

(53) $$\langle {\left(g_{\left(\mu )(\nu \right)}-{\tilde{g}}_{\left(\mu )(\nu \right)}\right)}^{2}\rangle \langle {\left(\Delta \pi_{\mu \nu }^{\left(q\right)}\right)}^{2}\rangle \geq \frac{\hbar^{2}}{4},$$

where the last one implies in turn the standard deviation (46). The two fluctuation inequalities (52) and (53) are referred to respectively as *first and second fluctuation Heisenberg inequalities.*

### 6.2. Another Set of “Effectively” Conjugate Variables

A second case of interest is represented by the set of quantum operators

(54) $$\left(A,B\right)=\left(\Delta g_{\mu \nu },\pi_{\mu \nu }^{\left(q\right)}\equiv -i\hbar \frac{\partial }{\partial g^{\mu \nu }}\right),$$

which are not conjugate variables per se but whose properties are “effectively” similar. Here, $\Delta g_{\mu \nu }$ denotes the stochastic displacement field characteristic of the Generalized Lagrangian Path (GLP) representation of CQG theory [7,8] which is related to the Lagrangian coordinate $g_{\mu \nu }$ by means of the transformation

(55) $$g_{\mu \nu }\left(s\right)=\Delta g_{\mu \nu }+G_{\mu \nu }\left(s\right).$$

Here, $G_{\mu \nu }\left(s\right)$ and $g_{\mu \nu }\left(s\right)$ represent the Lagrangian Path and the Generalized Lagrangian Path (GLP), respectively, being $\Delta g_{\mu \nu }$ a stochastic displacement field such that its covariant derivative is identically vanishing, namely, $\frac{d}{ds}\Delta g_{\mu \nu }=0$. A non-trivial Heisenberg inequality, similar but not equivalent to the previous one, nevertheless follows also in this case. To obtain it, it is sufficient to take into account the following exact differential and integral identities relating $\Delta g_{\mu \nu }$ to the Lagrangian coordinate $g_{\mu \nu }$ (see Appendix C in [8]), namely,

(56)∂Δgμν∂gαβ=p(s)δμαδνβ,(57)p(s)=1+2αL∫s0sds′a(s′)−1/2,

where $a\left(s\right)=\frac{1}{2}\left[a_{\left(o\right)}\left(s\right)+a_{\left(1\right)}\left(s\right)\right]\geq 0$ and $a_{\left(o\right)}\left(s\right),a_{\left(1\right)}\left(s\right)$ are suitable 4-scalar coefficients determined in the context of the GLP representation of CQG-theory [7,11]. Therefore, the commutator for the operators (A,B) becomes

(58) $$\left[\Delta g_{\left(\mu )(\nu \right)},\pi_{\mu \nu }^{\left(q\right)}\right]=i\hbar p\left(s\right),$$

where p(s) is a 4-scalar function prescribed in terms of Equation (57). In this case one obtains therefore the Heisenberg inequality

(59) $$\sigma_{\Delta g_{\left(\mu )(\nu \right)}}\sigma_{\pi_{\left(\mu )(\nu \right)}^{\left(q\right)}}\geq \frac{\hbar }{2}\tilde{p}\left(s\right).$$

As by construction $p(s_{0})=1$ while one can show that $\lim_{s\to \infty }p\left(s\right)=1$, one can expect $\tilde{p}\left(s\right)≳1$. Therefore, upon invoking the expectation value ${\tilde{\Delta g}}_{\mu \nu }={\hat{g}}_{\mu \nu }$, and noting that

(60) $$\tilde{p}\left(s\right)=\langle \psi |p(s)\psi \rangle \equiv F\left\{p(s)\right\},$$

one obtains

(61) $$\sigma_{\Delta g_{\mu \nu }}^{2}\equiv \langle {\left(\Delta g_{\left(\mu )(\nu \right)}-{\hat{g}}_{\left(\mu )(\nu \right)}\right)}^{2}\rangle \equiv \langle \psi \left|{\left(\Delta g_{\left(\mu )(\nu \right)}-{\hat{g}}_{\left(\mu )(\nu \right)}\right)}^{2}\psi \right.\rangle .$$

As a consequence invoking again the decomposition (49)–(51), the following stronger fluctuation Heisenberg inequality must hold,

(62) $$\langle {\left(\Delta g_{\left(\mu )(\nu \right)}-{\hat{g}}_{\left(\mu )(\nu \right)}\right)}^{2}\rangle {\langle {\left(\Delta \pi_{\mu \nu }\right)}^{2}\rangle }_{1}\geq \frac{\hbar^{2}}{4}\tilde{p}\left(s\right),$$

which implies

(63) $$\langle {\left(\Delta g_{\left(\mu )(\nu \right)}-{\hat{g}}_{\left(\mu )(\nu \right)}\right)}^{2}\rangle \langle {\left(\Delta \pi_{\mu \nu }^{\left(q\right)}\right)}^{2}\rangle \geq \frac{\hbar^{2}}{4}\tilde{p}\left(s\right).$$

The latter fluctuation inequality implies again the validity of the Heisenberg inequality for the corresponding standard deviations, which in this case is given by (59).

### 6.3. Proper-Time-Extended Canonical Momentum Inequality

The third relevant case is provided by the set of 4-scalar operators

(64) $$\left(A,B\right)=\left(s,p_{s}^{\left(q\right)}\equiv -i\hbar \frac{\partial }{\partial s}\right),$$

where *s* is the proper-time which parametrizes a local field geodetics $\left\{\left.r(s)\right|s\in \left[s_{0},s_{1}\right]\right\}$ and $p_{s}^{\left(q\right)}$ is the corresponding extended canonical momentum (see also Equation (36) in [2]). Manifestly both are Hermitian operators. In particular, setting now in Equation (28) $\psi_{a}^{*}\equiv \psi^{*}\left(s\right)$, $\psi_{b}\equiv p_{s}^{\left(q\right)}\psi \left(s\right)$, where $\psi \left(s\right)\equiv \psi (g,\hat{g}\left(r,s\right),r\left(s\right),s)$ denotes here an arbitrary element of the Hilbert space to be identified with a particular solution of the CQG-wave Equation (23), by construction it follows

(65) $$\begin{array}{ccc} \langle \langle \psi |p_{s}^{\left(q\right)}\psi \rangle \rangle & \equiv & F\left\{\int_{U_{g}}d\left(g\right)\psi^{*}\left(s\right)p_{s}^{\left(q\right)}\psi \left(s\right)\right\}\\ & = & F\left\{\int_{U_{g}}d\left(g\right)\left(p_{s}^{\left(q\right)}\psi^{*}\left(s\right)\right)\psi \left(s\right)\right\}=\langle \langle p_{s}^{\left(q\right)}\psi |\psi \rangle \rangle . \end{array}$$

The same relationship follows also by partial integration with respect to proper time *s*. In fact, $\langle \langle \psi |p_{s}^{\left(q\right)}\psi \rangle \rangle \equiv -i\hbar \langle \langle \psi |\frac{\partial }{\partial s}\psi \rangle \rangle$, and therefore integrating by parts $\langle \langle \psi |p_{s}^{\left(q\right)}\psi \rangle \rangle =i\hbar \langle \langle \frac{\partial }{\partial s}\psi |\psi \rangle \rangle =\langle \langle p_{s}^{\left(q\right)}\psi |\psi \rangle \rangle$. However, the product of Hermitian operators needs not be Hermitian. In particular direct evaluation shows that $\langle \langle \psi |sp_{s}^{\left(q\right)}\psi \rangle \rangle \neq \langle \langle sp_{s}^{\left(q\right)}\psi |\psi \rangle \rangle$. On the other hand, the commutator of $\left(s,p_{s}^{\left(q\right)}\right)$ is obviously

(66) $$\left[s,p_{s}^{\left(q\right)}\right]=i\hbar .$$

From the Robertson inequality (42) it therefore follows that

(67) $$\sigma_{s}\sigma_{p_{s}^{\left(q\right)}}\geq \frac{\hbar }{2},$$

which realizes *the proper-time-extended canonical momentum Heisenberg inequality. The corresponding quantum fluctuations* are therefore

(68)σs2≡ψ(s−s˜)2ψ,(69)σps(q)2≡ψps(q)−p˜s(q)2ψ,

with $\tilde{s}$, ${\tilde{p}}_{s}^{\left(q\right)}$ denoting the quantum expectation values for $\left(s,p_{s}^{\left(q\right)}\right)$. In particular, in case of a unitary solution one can show that

(70) $${\tilde{p}}_{s}^{\left(q\right)}=-{\tilde{H}}_{R}^{\left(q\right)},$$

where it follows

(71) $${\tilde{H}}_{R}^{\left(q\right)}=\frac{1}{2\alpha L}\left[{\langle \langle {\left(\Delta \pi_{{}_{\mu \nu }}\right)}^{2}\rangle \rangle }_{1}+{\langle \langle {\left(\Delta \pi_{{}_{\mu \nu }}\right)}^{2}\rangle \rangle }_{2}+{\tilde{\pi }}^{\mu \nu }{\tilde{\pi }}_{\mu \nu }\right].$$

Here, the quantum fluctuations ${\langle \langle {\left(\Delta \pi_{{}_{\mu \nu }}\right)}^{2}\rangle \rangle }_{1}$ and ${\langle \langle {\left(\Delta \pi_{{}_{\mu \nu }}\right)}^{2}\rangle \rangle }_{2}$ are given by Equations (50) and (51) while ${\tilde{\pi }}^{\mu \nu }$ is the momentum expectation value

(72) $${\tilde{\pi }}_{\mu \nu }=L\left\{\int_{U_{g}}d\left(g\right)\rho \frac{\partial S^{\left(q\right)}}{\partial g^{\mu \nu }}\right\}.$$

A remark is required regarding the prescription of the proper-time extrema which enter into the new definition of the scalar product (see Equation(28)) and affects the prescription of the fluctuations in Equations (68) and (69). In fact, in validity of the unitarity principle, letting

(73) $$s_{1}=s_{0}+\Delta s,$$

both $s_{0}$ and Δs remain in principle arbitrary, with Δs to be interpreted as the amplitude of proper time interval during which a quantum measurement is performed. As such it is obvious that the amplitude Δs cannot be arbitrarily small. In fact, since by construction $\sigma_{s}\equiv \frac{\Delta s}{2\sqrt{3}}$, it follows that the Heisenberg inequality (67) requires

(74) $$\Delta s\times \sigma_{p_{s}^{\left(q\right)}}\geq \hbar \sqrt{3}.$$

Let us briefly analyze the physical implications. In the context of the unitary representation of CQG-theory a number of notable consequences arises. The first one is that, thanks to manifest covariance, the inequality (74) holds in arbitrary GR-frames which belong to the same space-time $\left\{Q^{4},\hat{g}\right\}$, i.e., which are defined with respect to arbitrary coordinate systems mutually related by means of local point transformations $r^{\prime }=r^{\prime }\left(r\right)$. In fact, the inequality (74) states that the proper-length Δs, i.e., the proper-time amplitude of the quantum measurement, is effectively bounded from below, thus giving rise to a minimal length Δs being determined by the same inequality, i.e., in terms of the standard deviation of the conjugate quantum momentum operator, namely, $\sigma_{p_{s}^{\left(q\right)}}$. The consequence is therefore that the minimal length Δs is an invariant, i.e., it is a 4-scalar with respect to the background space-time of CQG-theory, i.e., $\left\{Q^{4},\hat{g}\right\}$. This means that Δs itself has an objective character (which is in a proper sense a mandatory requirement for a quantum observable), and therefore it has the same value when expressed again in arbitrary GR-frames belonging to $\left\{Q^{4},\hat{g}\right\}$. Finally, it is obvious the existence of the minimal invariant length Δs is not at variance with such a property and, therefore, the validity of CQG-theory.

These conclusions provide an important physical clue on the concept of minimal length, which is usually debated in the framework of phenomenological Generalized Uncertainty Principle (GUP) theories [39,40]. GUP models have been investigated as alternative quantum approaches to the study of quantum properties of classical black-hole solutions and related Hawking evaporation phenomena [41,42,43]. Unlike the canonical and manifestly covariant prescription of the present HIP originating from a quantization of the gravitational field, in the literature GUP proposals usually arise as heuristic non-canonical quantum theories, although similar generalized uncertainty relations can be inferred by non-commutative geometry [44] and string theory [45]. As a consequence, the minimal length predicted by GUP models might not be generally a 4-scalar, with the consequence of violation of the principle of manifest covariance. The present prescription suggests a possible way out to the problem, as the formalism of CQG-theory has a tensorial character and is therefore manifestly-covariant. Nevertheless, as a matter of consistency, precisely because of the canonical Hamiltonian structure of CQG-theory, the relation for the HIP determined above is distinguished from GUP models, as such relation does not contain any minimum length parameter on the rhs of the Heisenberg inequality.

## 7. Connection with the Deterministic Principle

As a final issue, the question which is addressed here is the relationship of HIP with the Deterministic Principle (DP) holding in the context of GR. This is known usually to be achieved by means of the semiclassical limit.The question is whether certain properties of the quantum wave solution, particularly the corresponding quantum probability density identified here with a Gaussian distribution in the *g*-space configuration space, can be used to investigate whether DP can be recovered by means of a suitable choice of the physical parameters.

One notices that in principle all integrals (i.e., all configuration space and proper-time integrals) which are involved in the calculations of the quantum fluctuations considered above can be performed analytically. We examine for this purpose separately the various types of quantum inequalities determined here.

Naively, the deterministic (i.e., classical) limit is often done by taking the limit ℏ→0, but here we employ a more sophisticated method, consisting of taking the limit of the semi-amplitude of the quantum Gaussian PDF in the Madelung representation to a delta-function up to the stochastic parameter. For this purpose, consider the shifted Gaussian quantum PDF $\rho_{G}$ of CQG-theory determined in [7]

(75) $$\rho_{G}\left(s\right)\equiv \rho_{G}\left(s\right)=K\exp\left\{-\frac{{\left(\Delta g-\hat{g}\left(s\right)\right)}^{2}}{r_{th}^{2}}\right\},$$

with $r_{th}^{2}$ being the constant dimensionless invariant semi-amplitude width of the Gaussian, while in short-notation the exponent ${\left(\Delta g-\hat{g}\right)}^{2}$ stands for the 4-scalar defined as ${\left(\Delta g-\hat{g}\right)}^{2}\equiv \left(\Delta g_{\mu \nu }-{\hat{g}}_{\mu \nu }\right)\left(\Delta g^{\mu \nu }-{\hat{g}}^{\mu \nu }\right)$. More precisely, $\hat{g}={\hat{g}}_{\mu \nu }$ is the background metric tensor and $\Delta g\equiv \Delta g_{\mu \nu }$ identifies the stochastic displacement symmetric tensor field associated with each stochastic quantum trajectory and such to have identically vanishing covariant derivative, namely, $\frac{D}{Ds}\Delta g_{\mu \nu }=0$. Given the solution (75) which is characterized by the semi-amplitude $r_{th}$, one has that in the limit $r_{th}\to 0$, the same quantum PDF $\rho_{G}$ tends necessarily to the limit

(76) $$\lim_{r_{th}\to 0}\rho_{G}\left(s\right)\equiv \lim_{r_{th}\to 0}\rho_{G}\left(s\right)=\delta \left(\Delta g-\hat{g}\left(s\right)\right),$$

which coincides with a Dirac-delta, i.e., which defines the so-called *deterministic limit*. Therefore such a limit should permit to recover the Deterministic Principle characteristic of GR. In other words, this means that in such a limit HIP and all related Heisenberg inequalities determined above should be satisfied identically. Let us proof that this is indeed the case.

Consider first the inequalities (52) and (53). Then one can show that the quantum fluctuation $\langle {\left(g_{\left(\mu )(\nu \right)}-{\tilde{g}}_{\left(\mu )(\nu \right)}\right)}^{2}\rangle$ is proportional to $r_{th}^{2}$. Analogous property holds for the quantum fluctuation $\langle {\left(\Delta g_{\left(\mu )(\nu \right)}-{\hat{g}}_{\left(\mu )(\nu \right)}\right)}^{2}\rangle$. Instead it is possible to prove that ${\langle {\left(\Delta \pi_{\mu \nu }\right)}^{2}\rangle }_{1}$, and therefore also $\langle {\left(\Delta \pi_{\mu \nu }^{\left(q\right)}\right)}^{2}\rangle$, are proportional to $1/r_{th}^{2}$. Therefore, the product of the corresponding standard deviations for the two sets of conjugate variables $\sigma_{g_{\left(\mu )(\nu \right)}}\sigma_{\pi_{\left(\mu )(\nu \right)}^{\left(q\right)}}$ and $\sigma_{\Delta g_{\left(\mu )(\nu \right)}}\sigma_{\pi_{\left(\mu )(\nu \right)}^{\left(q\right)}}$ both scale independent of the semi-amplitude $r_{th}^{2}$. Thus, it follows formally that the two inequalities (52) and (53) (and similarly (62) and (63)) remain valid also in the limit $r_{th}\to 0$ even if the limit functions of some of the factors may not exist. Nevertheless, the fact that $\langle {\left(g_{\left(\mu )(\nu \right)}-{\tilde{g}}_{\left(\mu )(\nu \right)}\right)}^{2}\rangle ∼r_{th}^{2}$ vanishes in such a limit, and consequently $\lim_{r_{th}\to 0}\sigma_{g_{\mu \nu }}^{2}=0$, means that the Lagrangian generalized coordinates are deterministic.

In a similar way by performing the analytic estimate of Δs and $\sigma_{p_{s}^{\left(q\right)}}$, one notices that while Δs is obviously constant, $\sigma_{p_{s}^{\left(q\right)}}$ can be shown to include an additive term proportional to the factor $1/r_{th}^{2}$, namely, it scales with respect to the Gaussian semi-amplitude $r_{th}^{2}$ as

(77) $$\sigma_{p_{s}^{\left(q\right)}}∼\frac{1}{r_{th}^{2}}.$$

Therefore, the product of standard deviations for the set of conjugate variables (64) is such that similarly

(78) $$\sigma_{s}\sigma_{p_{s}^{\left(q\right)}}∼\frac{1}{r_{th}^{2}}.$$

This feature distinguishes the proper-time conjugate variables from the previous two sets of variables and implies that, in the limit $r_{th}\to 0$, $\sigma_{p_{s}^{\left(q\right)}}$ diverges. Thus, the inequality (67) is necessarily satisfied identically in the deterministic limit. This means, therefore, that

(79) $$\lim_{r_{th}\to 0}\sigma_{p_{s}^{\left(q\right)}}=\lim_{r_{th}\to 0}\sigma_{s}\sigma_{p_{s}^{\left(q\right)}}=+\infty ,$$

which implies, in turn, that in this case the inequality (74) is also identically satisfied.

To conclude we should mention, however, that actually $r_{th}$ cannot be regarded as a free parameter. In this connection we mention, in fact, that in [11] the numerical value of $r_{th}$ was prescribed to be $r_{th}∼O\left(1\right)$ to fit the experimentally observed estimate for the cosmological constant. Which means that the previous Heisenberg inequalities are expected to place meaningful constraints (i.e., lower bounds) on the related standard deviations.

## 8. Conclusions

In this paper, it has been proved that in quantum gravity, just as in quantum mechanics and quantum field theory, the role of quantum uncertainty, and in particular of the Heisenberg Indeterminacy Principle (HIP), is a fundamental one. For this purpose the point of view of manifestly-covariant formulation of quantum gravity has been adopted, which as discussed above represents a possible relativistic covariant generalization of the Wheeler–DeWitt equation.

Our main claim is that also in Quantum Gravity the validity of HIP can be viewed as a paradigm of a quantum theory in itself. The indeterminacy (“uncertainty” in Heisenberg words) arises because of the concept of quantum measurement and the non-deterministic, i.e., stochastic, nature of quantum observables. This means that their quantum expectation values must exhibit their non-deterministic nature in terms of suitable inequalities, the so-called Heisenberg inequalities. Such inequalities, however, to make sense from the physical standpoint, must necessarily be expressed in manifest covariant form. In other words, the same inequalities must necessarily be endowed with tensor properties with respect to the background classical space-time. This means that the underlying quantum theory itself too should be manifestly covariant, namely be expressed in 4-tensor form. This conclusion, however, is not unexpected and has been discussed exhaustively before [27].

In this treatment aspects of the Heisenberg uncertainty principle arising in the context of covariant quantum gravity have been investigated. The purpose of the investigation has been to extend the Heisenberg Indeterminacy Principle to the case of the proper-time-conjugate momentum inequality. Based on the unitary representation of covariant quantum gravity the problem has been posed to determine the corresponding scalar product for the Hilbert-space settings. As a result, a general proof of the Heisenberg Principle has been reached. Besides the (customary) case of conjugate canonical variables, a further Heisenberg inequality exists, namely, the proper-time-extended canonical momentum inequality which relates the standard deviations of the quantum proper-time and of the corresponding conjugate extended quantum canonical momentum operator.

The inequality is analogous to the coordinate time and energy inequality holding both in non relativistic and relativistic quantum mechanics. However, the result highlights remarkable physical aspects of covariant quantum gravity theory:

- The first one is that, thanks to manifest covariance, all Heisenberg inequalities, including the one involving proper-time and its conjugate extended canonical momentum hold in arbitrary GR-frames.
- The proper-time amplitude of the quantum measurement is effectively bounded from below.
- The bounded length is an invariant, i.e., it is a 4-scalar with respect to the background space-time of CQG-theory, so that validity of CQG-theory is warranted.

Finally, the conditions of validity of the resulting Heisenberg inequalities have been discussed together with their deterministic limits, showing that they recover their correct classical, i.e., GR limits.

To conclude, we should mention that the theory of Heisenberg inequalities is susceptible of a wide range of theoretical-physics related applications and generalizations. One such possible development includes the treatment of a non-unitary representation of CQG-theory. This task will be undertaken elsewhere.

## Appendix A. Quantum Hamiltonian Operator

In this appendix, we recall the relevant definitions pertaining to the quantum Hamiltonian operator $H_{R}^{\left(q\right)}$ which are based on the works in [2,3,4,5,6,7,8]. Thus, denoting $g_{\mu \nu }^{\left(q\right)}\equiv g_{\mu \nu }$, $\pi^{\left(q\right)}\equiv -i\hbar \frac{\partial }{\partial g^{\mu \nu }}$, respectively, the canonical generalized coordinates and conjugate quantum canonical momenta, the quantum Hamiltonian operator $H_{R}^{\left(q\right)}$ is taken of the form

(A1)HR(q)≡TR(q)(π(q),g^)+V,(A2)TR(q)(π(q),g^)=12αL−iℏ∂∂gμν−iℏ∂∂gμν,

where $T_{R}^{\left(q\right)}$ and *V* denote the quantum effective kinetic energy operator and the normalized effective potential density, respectively. Here, *V* takes the form (see the works in [5,6])

(A3) $$\left\{\begin{array}{c} V\left(g,\hat{g},r,s\right)\equiv V_{o}\left(g,\hat{g},r,s\right)+V_{F}\left(g,\hat{g},r,s\right),\\ V_{o}\equiv h\alpha L\left[g^{\mu \nu }{\hat{R}}_{\mu \nu }-2\Lambda \right],\\ V_{F}\left(g,\hat{g},r\right)\equiv hL_{F}\left(g,\hat{g},r\right), \end{array}\right.$$

with $V_{o}$ and $V_{F}$ representing the vacuum and external field contributions. The rest of the notations is as follows. First, ${\hat{R}}_{\mu \nu }\equiv R_{\mu \nu }\left(\hat{g}\right)$ and Λ identify respectively the background Ricci tensor and the cosmological constant, $L_{F}$ being associated with possible non-vanishing stress-energy tensor. In addition, *h* is the variational weight-factor

(A4) $$h\left(g,\hat{g}\left(r,s\right)\right)=2-\frac{1}{4}g^{\alpha \beta }g^{\mu \nu }{\hat{g}}_{\alpha \mu }\left(r,s\right){\hat{g}}_{\beta \nu }\left(r,s\right).$$

Finally, *L* and α are suitable dimensional constants, i.e., suitable 4-scalars both identified according to the treatment given in [6].

## Appendix B. Effective Hamiltonian Operator

In terns of the quantum phase-function

(A5) $$S^{\left(q\right)}\left(g,\hat{g},r,s\right)\equiv S^{\left(q\right)}\left(g,\hat{g},r,s;P\right),$$

the quantum 4-tensor velocity field is defined as

(A6) $$V_{\mu \nu }=\frac{1}{\alpha L}\frac{\partial S^{\left(q\right)}}{\partial g^{\mu \nu }}.$$

Similarly, the effective quantum Hamiltonian density takes the form

(A7) $$H_{R}=\frac{1}{2\alpha L}\frac{\partial S^{\left(q\right)}}{\partial g^{\mu \nu }}\frac{\partial S^{\left(q\right)}}{\partial g_{\mu \nu }}+V_{QM}+V,$$

with V≡V(g,s) being the effective potential defined according to Equation (A3) (see Appendix A) and $V_{QM}\equiv V_{QM}\left(g,s\right)$ being the Bohm effective quantum potential [46] of CQG-theory. The latter, in particular, recovers the customary expression

(A8) $$V_{QM}\equiv \frac{\hbar^{2}}{8\alpha L}\frac{\partial \ln\rho }{\partial g^{\mu \nu }}\frac{\partial \ln\rho }{\partial g_{\mu \nu }}-\frac{\hbar^{2}}{4\alpha L}\frac{\partial^{2}\rho }{\rho \partial g_{\mu \nu }\partial g^{\mu \nu }},$$

with $\rho =\rho (g,\hat{g},r,s)$ being determined by the quantum continuity equation. Thus, the prescription of $V_{QM}$ actually depends as in [7,8] on the precise determination of particular solutions of the same equation to be based once again on the so-called Generalized Lagrangian Path (GLP) representation of CQG-theory and of its CQG-wave equation.
